# Retraction: MALAT1 predicts poor survival in osteosarcoma patients and promotes cell metastasis through associating with EZH2

**DOI:** 10.18632/oncotarget.28896

**Published:** 2026-06-17

**Authors:** Yanqing Huo, Qingbo Li, Xiqian Wang, Xiejia Jiao, Jiachun Zheng, Zhiqiang Li, Xiaohan Pan

**Affiliations:** ^1^Department of Orthopedics, The Second Hospital of Shandong University, Jinan, 250133, Shandong Province, China; ^2^Department of Health Management, The Second Hospital of Shandong University, Jinan, 250133, Shandong Province, China

**This article has been retracted:** Oncotarget has concluded its investigation of this paper, and the following issues were discovered:

- Figure 4D: The E-cadherin western blot is a duplicate of Figure 4B from a previously submitted article [1]. While the articles share no authors, they originated from the same institution. Additionally, the β-actin blot is a duplicate of the β-actin blot in Figure 4E of an unrelated paper [2].
- Figure 4C: The invasion assay image for MG-63/si-NC overlaps with the Figure 7D invasion image for MG-63/pMALAT1 + si-EZH2.
- Figure 7D: The invasion image for SAOS-2/pMALAT1 was found in a later publication [3] as the HT29/Control in Figure 1D.

The authors did not respond to requests for comment or provide the original data. Consequently, the editorial decision has been made to retract this paper. Oncotarget has reached out to all authors to notify them of this retraction but has received no response.

Original article: Oncotarget. 2017; 8:46993–47006. 46993-47006. https://doi.org/10.18632/oncotarget.16551
